# Exhaled and arterial levels of endothelin-1 are increased and correlate with pulmonary systolic pressure in COPD with pulmonary hypertension

**DOI:** 10.1186/1471-2466-8-20

**Published:** 2008-09-26

**Authors:** Pierluigi Carratu, Cristina Scoditti, Mauro Maniscalco, Teresa Maria Seccia, Giuseppe Di Gioia, Felice Gadaleta, Rosa Angela Cardone, Silvano Dragonieri, Paola Pierucci, Antonio Spanevello, Onofrio Resta

**Affiliations:** 1 Institute of Pulmonary Disease, University of Bari, Italy; 2 Institute of Pulmonary Disease, University of Naples, Italy; 3 Department of General and Environmental Physiology, University of Bari, Italy; 4 Institute of Pulmonary Disease, University of Foggia, Italy

## Abstract

**Background:**

Endothelin-1 (ET-1) and Nitric Oxide (NO) are crucial mediators for establishing pulmonary artery hypertension (PAH). We tested the hypothesis that their imbalance might also occur in COPD patients with PAH.

**Methods:**

The aims of the study were to measure exhaled breath condensate (EBC) and circulating levels of ET-1, as well as exhaled NO (FENO) levels by, respectively, a specific enzyme immunoassay kit, and by chemiluminescence analysis in 3 groups of subjects: COPD with PAH (12), COPD only (36), and healthy individuals (15). In order to evaluate pulmonary-artery systolic pressure (PaPs), all COPD patients underwent Echo-Doppler assessment.

**Results:**

Significantly increased exhaled and circulating levels of ET-1 were found in COPD with PAH compared to both COPD (p < 0.0001) only, and healthy controls (p < 0.0001). In COPD with PAH, linear regression analysis showed good correlation between ET-1 in EBC and PaPs (r = 0.621; p = 0.031), and between arterial levels of ET-1 and PaPs (r = 0.648; p = 0.022), while arterial levels of ET-1 inversely correlated with FEV_1_%, (r = -0.59, p = 0.043), and PaPs negatively correlated to PaO_2 _(r = -0.618; p = 0.032). Significantly reduced levels of FENO were found in COPD associated with PAH, compared to COPD only (22.92 ± 11.38 vs.35.07 ± 17.53 ppb; p = 0.03). Thus, we observed an imbalanced output in the breath between ET-1 and NO, as expression of pulmonary endothelium and epithelium impairment, in COPD with PAH compared to COPD only. Whether this imbalance is an early cause or result of PAH due to COPD is still unknown and deserves further investigations.

## Background

Chronic obstructive pulmonary disease (COPD) is the fourth leading cause of morbidity and mortality in developed countries. Pulmonary artery hypertension (PAH), frequently observed in patients with advanced COPD, is considered a predictor of worse outcome [1,2]. PAH in COPD is mainly the result of hypoxic pulmonary vasoconstriction [3], however it has been recently shown also induced by pulmonary arterial walls remodelling, with aberrant intimal changes [4,5]. Further evidences suggest that the initial event in the natural history of pulmonary hypertension due to COPD is the lesion of pulmonary endothelium by cigarette-smoke products [6,7], leading to critical changes in the expression of vascular mediators [7,8], which are responsible for impairment of endothelial function. Among a wide spectrum of vasoactive mediators, endothelin-1 (ET-1) plays a pivotal role in exerting vasoconstriction [9], bronchoconstriction [10], vascular [11] and airway [12] cells proliferation, via ET_A _and ET_B _receptors [13,14]. Increased expression of ET-1, it has been identified in the vessels of patients with IPAH [15]. Plasma [16,17], induced sputum [18], and urinary [17] levels of ET-1 are augmented in patients with COPD; however, there are still contrasting data about circulating ET-1 levels in COPD patients with PAH [19-21]. In addition, no data are yet available on ET-1 levels in exhaled breath condensate of COPD patients.

Nitric oxide (NO), produced by endothelial cells, is the central stimulus for releasing and dilating pulmonary arterial vasculature [22]. NO production is oxygen dependent and lack of NO synthesis, under hypoxic conditions such as COPD, contributes to chronic hypoxic pulmonary vasoconstriction [23], leading to pulmonary artery hypertension [24]. It is now appreciated that NO release is impaired in the pulmonary vasculature of COPD patients [25], while exhaled NO levels, increased in COPD [26], are significantly reduced in COPD associated with PAH [27]. An imbalance between the excretion of vascular mediators has been shown important for developing idiopathic pulmonary hypertension [8] and might be also responsible in promoting secondary PAH [8]. We hypothesized a pathological dysregulation of exhaled output between ET-1 and NO in COPD with PAH. Despite several issues about reproducibility, variability and sensitivity, measurement of different biological markers in COPD is currently a useful assay in assessing disease pathogenesis, predicting progression, and monitoring adequate therapies [28]. The aims of the present study were to investigate exhaled breath condensate (EBC) and circulating levels of ET-1, as well as the exhaled NO (FENO) in COPD only, in COPD with pulmonary artery hypertension, and in a group of healthy subjects. We also examined whether concentrations of exhaled biomarkers, or circulating ET-1 levels were related to the severity of disease, as defined by lung function, or pulmonary artery pressure.

## Methods

The Institutional Review and the Ethical Boards of the University of Bari approved protocols and all patients signed informed consent before participating in this study.

### Patient population

Sixty-three subjects were enrolled into the study at outpatient clinic of Institutes of pulmonary disease, University of Bari, from March 2004 to January 2007. Subjects were divided into three groups on the basis of pathological characteristics. The first group consisted of 12 patients with COPD (stage III and IV), diagnosed according to the American Thoracic Society (ATS) criteria [29] (11 males, mean age 70.8 ± 6.7 years), associated with PAH. All patients were ex smokers (mean pack-years, 26 ± 7) without a history of atopy and with normal IgE levels. Five patients had been receiving long-term domiciliary oxygen for at least 6 consecutive months.

A second group included 36 patients with COPD, diagnosed according to the American Thoracic Society (ATS) criteria (30). All patients (32 males, mean age 67.6 ± 9.2 years) were ex smokers (mean pack-years, 25 ± 6) without a history of atopy and with normal IgE levels; six patients had been receiving long-term domiciliary oxygen for at least 6 consecutive months. 15 healthy non-smoking, non-atopic subjects (9 males, mean age 58 ± 9.2 years) were enrolled as controls. Patients with other organ failure, cancer, or inability to cooperate were excluded from the study. At the time of inclusion into the study, all the patients were in stable condition, and free from respiratory tract infection and/or acute exacerbation in the preceding 6 weeks. All of the patients were receiving their regular treatment with inhaled bronchodilators, but none was receiving systemic or inhaled steroids. No change in medical therapy was made in the week prior to the study.

### Pulmonary function test and arterial blood gas analysis

Pulmonary function tests were performed in the pulmonary function laboratory of our Institutes at admission to the study. Static and dynamic lung volumes were measured by means of a constant-volume body plethysmograph (MasterScreen Body, Jaeger, Germany) and according to the guidelines of the American Thoracic Society (ATS) [30]. The best of three reproducible values was expressed as a percentage of the predicted normal value.

Arterial blood for the analysis of gases during room air breathing was drawn in all patients by radial artery in the supine position and after a 30 minute rest period. PaO_2_, PaCO_2 _and pH were measured in a blood gas analyzer (Model 1312; Instrumentation Laboratory; Milan, Italy).

### Echo-Doppler assessment

All patients of groups 1-2-3 were investigated using real-time, phased array, two-dimensional Doppler (2-D) echocardiography (CFM 750 CV 2.5 or 3.25 MHz transducer; GE Vingmed, Milan, Italy). The examinations were taken on patients in a semirecumbent left lateral position, and images were taken from subxiphoid, parasternal, and apical views. The mean value of three measurements was considered. Tricuspid valve regurgitation pressure was identified by color flow mapping, then, maximal pressure gradient between right ventricular and right atrial was obtained using the continuous wave Doppler on the guidance of the color Doppler signal of the tricuspid regurgitation. Peak pressure gradient measurement was estimated by means of a simplified Bernoulli equation [31]. Pulmonary-artery systolic pressure (PaPs) was calculated by adding the Bernoulli derived pressure gradient to an assumed right atrial pressure of 10 mmHg. A PaPs ≥ 35 mmHg was considered as cut-off.

### Measurement of ET-1 levels

ET-1 was evaluated in the (EBC) as well as in arterial and venous blood of all the subjects. ET-1 was obtained by using a condenser non-invasively to collect the non-gaseous components of the expiratory air (EcoScreen: Jaeger, Würzburg, Germany). Subjects were instructed to breathe through a mouthpiece and a two-way, non-rebreathing valve, which also served as a saliva trap, in order to avoid contamination. They were asked to breathe at tidal volume, wearing a nose clip, for a period of 10 min. The condensate (at least 1 ml) was transferred to centrifuges tubes and immediately stored at -80°C. Peripheral venous blood samples were obtained, in all subjects, from an antecubital vein after at least 30 min seated rest. Venous blood samples were collected in plastic tube containing ethylenediamine tetraacetic acid (EDTA), centrifuged at 5000*g for 10 min and plasma was frozen and stored at -80°C until assay. Arterial blood gas analyses were performed in all subjects, while breathing room air, by radial artery in the supine position and after a 30 minute rest period. A specific enzyme immunoassay (EIA) kit (Cayman Chemical, Ann Arbor, Mich., USA) was used to measure ET-1 concentrations in the breath condensate and in blood samples. The specificity of the test for ET-1 was 100%, and the cut-off of the assay was considered a concentration ≥ 1.5 pg/ml. The reproducibility of repeated ET-1 exhaled condensate measurements was assessed by the Bland and Altman method [32].

### NO measurement

Exhaled NO was measured by a chemiluminescence analyzer (NOA Tm280; Sievers Instruments Inc., Boulder, CO, USA) by the on-line single-breath technique, according to the ATS and ERS recommendations [33]. Subjects were asked to perform a single slow exhalation starting from total lung capacity through a mouthpiece against a resistance of 16 cm H_2_O under a visual biofeedback, to maintain a 50 ml second (s) steady flow. Subjects were at rest, sitting down, having refrained from eating and exercise for at least 2 hours, and breathed filtered NO-free air (air filter AFL 01410; Sievers Instruments Inc.) without a nose-clip before the single exhalation maneuver. Ambient air NO was recorded before and after each test. FENO levels were measured at the plateau of the end exhaled reading and expressed as parts per billion (ppb). At least three measurements that varied by <10% or two measurements that varied by <5% were recorded. Mean values of the respective plateaux were evaluated.

### Six minutes walking test

The 6-min walking distance (6 MWD) test was performed, at admission to the study, in 6 COPD with PAH, according to standard method [34].

### Statistical analysis

Data are presented as mean value ± standard deviation (SD). As the data presented normal distribution (Kolmogorov-Smirnov test), parametric tests were used for statistical analysis. Analysis of variance (ANOVA) was used to perform comparisons between groups. Relationships between patients' characteristics were evaluated using Pearson correlation coefficients. Comparisons of means ± SD among the groups were made by an unpaired t-test. Significance was established at a p-value < 0.05. StatView version 5.0 (SAS Institute, Cary, N.C., USA) was used for the statistical analysis. Both, correlations between clinical parameters and exhaled or circulating levels of ET-1 presented as scattered plots, and exhaled levels of ET-1 and NO expressed as bar graphs, were made by using KaleidaGraph program (Version 3.02, Synergy soft-ware, PA, USA).

## Results

Demographic and clinical characteristics of 63 individuals recruited are presented in table 1. Endothelin-1 was detectable in the exhaled breath as well as in the blood of all subjects. The reproducibility of repeated ET-1 measurements was quite good, and was assessed by the Bland and Altman method [32] in six patients (mean difference 0.62 ± 1.214). NO was detectable in the exhaled of all individuals. All COPD patients were investigated with Echo-Doppler and then assigned to one of two groups according to the level of PaPs, using a cut-off of 35 mmHg. Those with a PaPs ≥ 35 mmHg (Group 1: N = 12; FEV_1_% 39.35 ± 11.45; PaPs 51.08 ± 12.21 mmHg), and those with a PaPs < 35 mmHg (Group 2: N = 36; FEV_1_% 48.13 ± 18.04; PaPs 26.05 ± 3.39 mmHg).

**Table 1:** Demographic and clinical characteristics of 63 individuals recruited classified in 3 groups.

	Group ofCOPD+PAH (n = 12)	Group of COPD (n = 36)	Group of HS (n = 15)
Sex, M/F	11/1	32/4	9/6
Age, Years	70.8 (6.7)	67.6 (9.2)	58 (9.2)
Smoking, pack-years	27 (7)	25 (6)	0
FEV_1_%	39.35 (11.45)	48.13 (18.04)	98.05 (11.65)
FVC%	64.89 (14.90)	69.39 (19.1)	95.07 (13.34)
FEV_1_/FVC %	46.95 (6.81)	54.27 (12.46)	101.37 (8.5)
RV%	151 (38)	143 (32)	108 (11)
PaO_2 _(mmHg)	60.83 (4.76)	64.58 (6.16)	97.2 (0.7)
PaCO_2 _(mmHg)	39.4 (4.27)	41.78 (5.01)	38.4 (1.5)
PaPs (mmHg)	51.08 (12.2)*	26.05 (3.39)	NA
ET-1 EBC (pg/ml)	22.36 (4)	7.25 (0.23)	5.24 (0.47)
ET-1 art (pg/ml)	12.41 (2.13)	7.22 (0.21)	0.84 (0.37)
ET-1 ven (pg/ml)	14.53 (2.76)	7.29 (0.16)	1 (0.42)
FENO (ppb)	22.92 (11.38)	35.07 (17.53)	20.56 (3.67)
6MWD	383 (34)^§^	NA	NA

Pulmonary function, arterial blood gases, pulmonary-artery systolic pressure, exhaled breath condensate counts and circulating levels are expressed in table 1. Statistical significant difference was found between groups 1 and 2 in pulmonary-artery systolic pressure, PaPs (p < 0.0001).

*Definition of abbreviation*s

EBC: Exhaled Breath Condensate, ET-1: Endothelin-1, FENO: Exhaled Nitric Oxide, FEV_1_: Forced Expiratory Volume in the first second, FVC: Forced Vital Capacity, HS: Healthy Subjects, NO: Nitric Oxide, PAH: Pulmonary Artery Hypertension, RV: Residual Volume, PaPs: Pulmonary-artery systolic pressure, 6MWD: 6-minutes walking distance

Data are presented as mean (± SD)

NA = not applicable

§ n = 6 patients

* Significant difference compared to COPD group: p < 0.001

### Group of COPD with PAH

**Figure 1:**
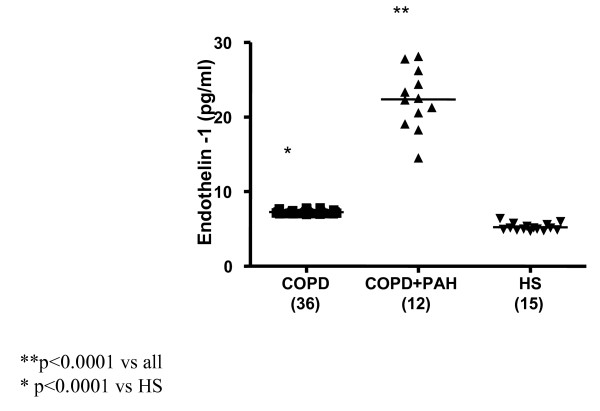
**Endothelin-1 (expressed as pg/ml) concentration in EBC of the 3 groups: COPD (36), COPD + PAH (12), and healthy subjects (HS) (15).** Significance was established at a p value < 0.05. The horizontal lines represent median value. ET-1 in EBC was increased in all COPD patients compared to control group (p < 0.0001). In COPD+PAH group, ET-1 levels were significantly increased compared to both the COPD only and the control group (p < 0.0001).

**Figure 2:**
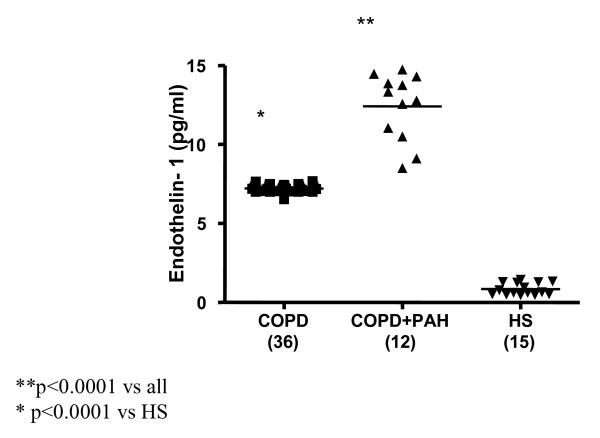
**Endothelin-1 (expressed as pg/ml) levels in the arterial blood of the 3 groups: COPD (36), COPD + PAH (12), and healthy subjects (HS) (15).** Significance was established at a p value < 0.05. The horizontal lines represent median value. ET-1 in arterial sample was increased in all COPD patients compared to control group (p < 0.0001). In COPD+PAH group, ET-1 levels were significantly increased compared to both the COPD only and healthy controls (p < 0.0001).

**Figure 3:**
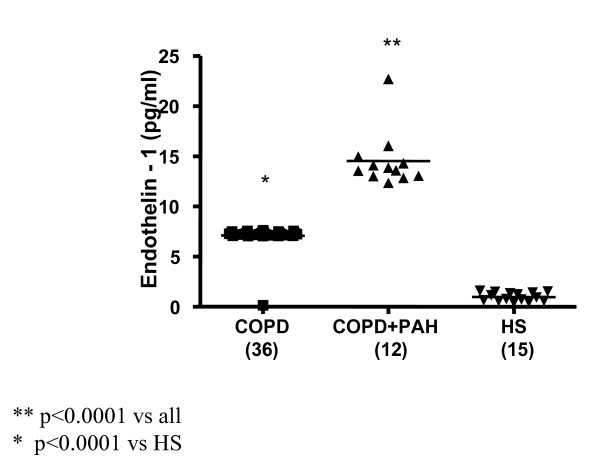
**Endothelin-1 (expressed as pg/ml) concentration in the venous blood of the 3 groups: COPD (36), COPD + PAH (12), and healthy subjects (HS) (15).** Significance was established at a p value < 0.05. The horizontal lines represent median value. ET-1 in venous sample was increased in all COPD patients compared to control group (p < 0.0001). In COPD+PAH group, ET-1 levels were significantly increased compared to both the COPD only and the controls (p < 0.0001).

**Figure 4:**
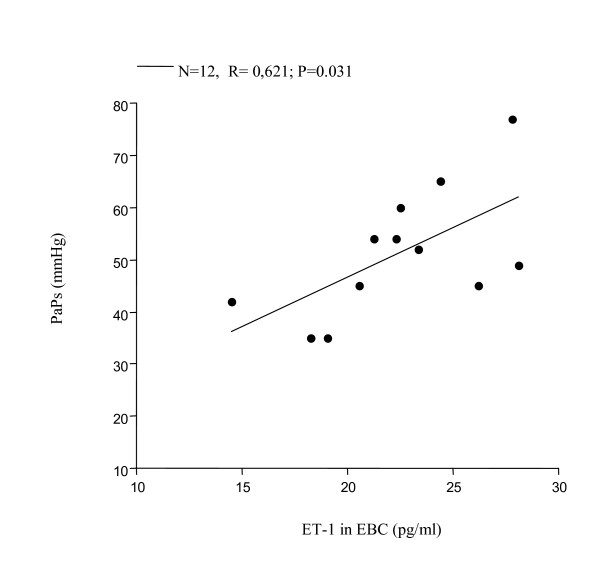
COPD+PAH: linear regression shows good correlation between ET-1 levels in the EBC and PaPs (N = 12; r = 0.621; p = 0.031).

**Figure 5:**
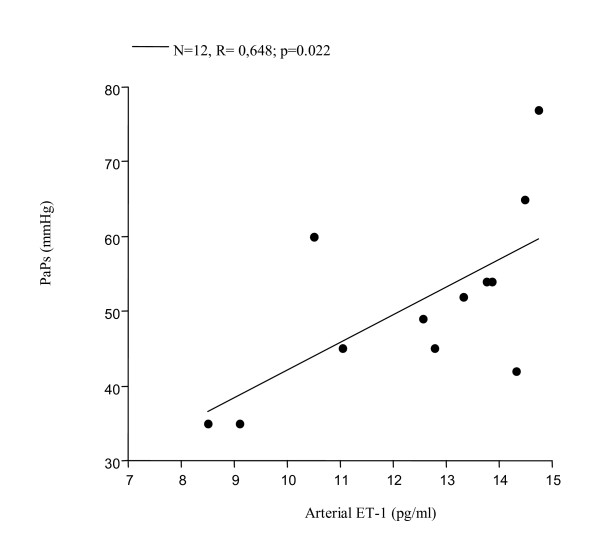
COPD+PAH: positive correlation between arterial blood levels of ET-1 and PaPs (N = 12, r = 0.65; p = 0.022).

**Figure 6:**
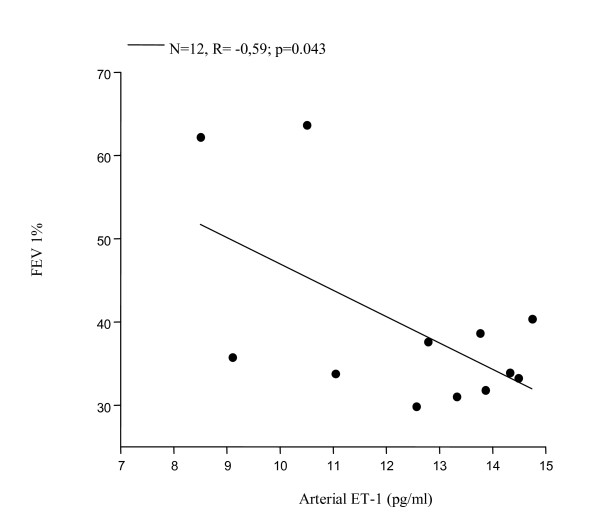
COPD+PAH: negative correlation between FEV_1_% and arterial blood levels of ET-1 (N = 12, r = -0.59, p = 0.043).

**Figure 7:**
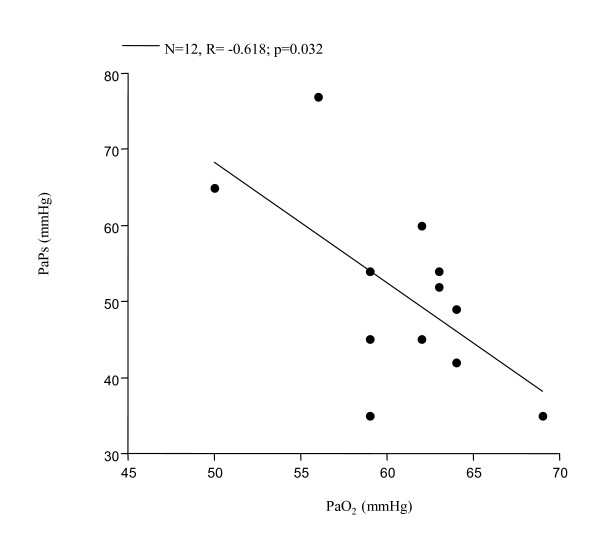
COPD+PAH: negative correlation between PaPs and PaO_2 _(N = 12, r = -0.618; p = 0.032).

Significantly increased levels of ET-1 in EBC were found in all 12 patients with PAH associated with COPD (FEV_1_% 39.35 ± 11.45, FEV_1_/FVC% 46.95 ± 6.81, PaPs ≥ 35 mmHg) (group 1) compared to both COPD only (group 2) (22.36 ± 4 vs 7.25 ± 0.23 pg/ml; p < 0.0001) and to the control subjects (group 3) (5.24 ± 0.47 pg/ml; p < 0.0001) (Figure 1). Higher circulating levels of ET-1 both in the arterial and in the venous sample were found in group 1 compared to all other groups. Arterial levels were increased as compared to group 2 (12.41 ± 2.13 vs 7.22 ± 0.21 pg/ml; p < 0.0001) and to the controls (0.84 ± 0.37 pg/ml; p < 0.0001) (Figure 2). We also observed increased levels of ET-1 in the venous blood statistically significant compared to both group 2 (14.53 ± 2.76 vs 7.29 ± 0.16 pg/ml; p < 0.0001), and to the control group (1 ± 0.42 pg/ml; p < 0.0001) (Figure 3). Thus, significantly over-expression of ET-1 was found in COPD with PAH compared to COPD only. With regards to disease severity, in COPD with PAH, Pearson correlation analysis showed a good relationship between ET-1 in EBC and pulmonary-artery systolic pressure (PaPs) (r = 0.621; p = 0.031) (Figure 4); A positive correlation was also observed between arterial blood levels of ET-1 and PaPs (r = 0.648; p = 0.022) (Figure 5), while the arterial blood levels of ET-1 inversely correlated to the FEV_1_% of the group 1 (r = -0.59, p = 0.043) (Figure 6). Furthermore, PaPs inversely correlated with PaO_2 _in this group of patients (r = -0.618; p = 0.032) (Figure 7). ET-1 levels in venous blood, although higher than arterial levels (14.53 ± 2.76 vs. 12.41 ± 2.13), did not correlate with any parameter considered in this group of patients.

**Figure 8:**
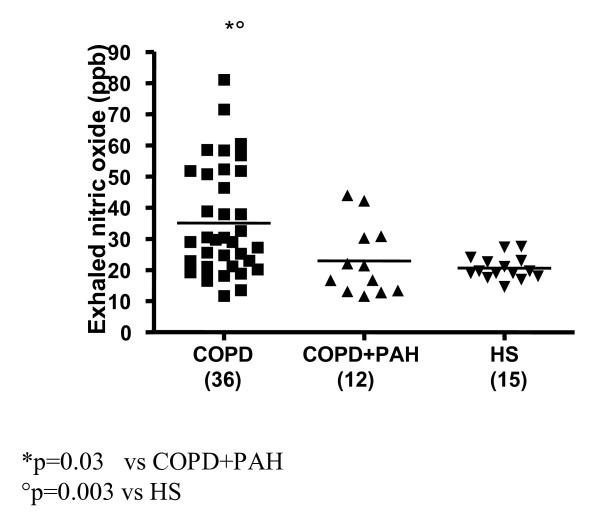
**Exhaled nitric oxide concentrations (FENO), expressed as ppm, in 3 groups: COPD (36), COPD+PAH (12), and healthy subjects (HS) (15). **Significance was established at a p value < 0.05. The horizontal lines represent median value. FENO levels were significantly higher in COPD only, compared to both the COPD with PAH (p = 0.03), and the controls (p = 0.003). There was no significant relationship between FENO levels in COPD+PAH compared to the control group levels (p = 0.45).

**Figure 9:**
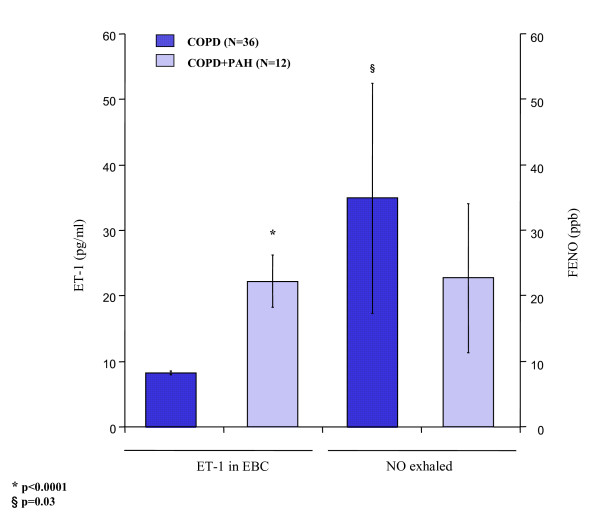
**An imbalance between exhaled condensate ET-1 concentrations and exhaled nitric oxide concentrations (FENO) in COPD (36), and in COPD+PAH (12).** In COPD+PAH group, ET-1 levels were significantly increased compared to the COPD only (p < 0.0001). FENO levels were significantly higher in COPD only, compared to both the COPD with PAH (p = 0.03).

In COPD patients with PAH, we found significantly reduced levels of FENO compared to COPD only (22.92 ± 11.38 vs.35.07 ± 17.53 ppb; p = 0.03) (Figure 8). These levels were not different compared to the FENO levels of the control group (20.56 ± 3.67 ppb; p = 0.45) (Figure 8). An imbalanced output in the breath between ET-1 and NO was seen in this group of patients. As shown in figure 9, ET-1 levels were significantly increased in COPD with PAH compared to COPD only (p < 0.0001), while FENO levels in group 1 were significantly lower than group 2 levels (p = 0.03). FENO levels did not significantly correlate with any clinical feature.

### Group of COPD only

In 36 COPD patients (FEV_1_% 48.13 ± 18.04, FEV_1_/FVC% 54.27 ± 12.46, PaPs <35 mmHg) ET-1 levels in EBC were significantly higher than control group levels (5.24 ± 0.47 pg/ml; p < 0.0001) (Figure 1). Increased concentrations of ET-1 in the arterial, as well as in the venous blood, compared to the control group, were found in group 2 (7.22 ± 0.21 vs. 0.84 ± 0.37 pg/ml; p < 0.0001; 7.29 ± 0.16 pg/ml vs. 1.00 ± 0.42 pg/ml; p < 0.0001) (Figures 2, 3). FENO levels in COPD were statistically significant higher than control group levels (35.07 ± 17.53 vs. 20.56 ± 3.67 ppb; p = 0.003), (Figure 8). Two COPD patients showed higher NO levels, similar to those detected in asthma and not in COPD, but we excluded that those levels were related to an atopic status, on the basis of the IgE total concentration. ET-1 and NO levels did not significantly correlate to any baseline characteristic in this group of patients.

## Discussion

In the natural history of COPD, right heart failure leading to increased pulmonary artery pressure is a common finding [35,36]. PAH, in this scenario, is likely due to a primary hypoxic vasoconstriction associated to the direct toxic effect of tobacco smoke into the intrapulmonary vessels, with abnormal production of substances that control vasoconstriction, vasodilatation, and vascular cell proliferation, ultimately leading to extensive pulmonary artery remodelling [2,37]. Early detection of an imbalanced output between essential mediators, such as endothelin-1 and NO, might be a novel approach to rapidly investigate and eventually treat this critical disease. The present study was designed to investigate endothelin-1 and NO concentration in the exhaled breath, as well as circulating levels of ET-1 in COPD patients with or without pulmonary hypertension. Our findings show that endothelin-1 levels in EBC are increased in all COPD patients. In addition, we observed ET-1 levels in EBC significantly higher in COPD associated with PAH, compared to COPD only. Several previous studies detected elevated levels of ET-1 in EBC in interstitial pulmonary disease [38], in non small cell lung cancer patients (NSCLC) [39], and in unstable bronchial asthma [40], similar to those observed in our COPD patients, while in the present, we found significantly higher EBC levels of ET-1 in COPD with PAH. Carpagnano and colleagues hypothesized that ET-1, in pathological conditions (interstitial pulmonary disease and NSCLC), could be in part produced by airway epithelium itself [38,39], while the reason of an exaggerated ET-1 release in EBC in COPD with PAH is still unknown. However, it appears that ET-1, primary produced by the pulmonary endothelium, could be in part generated by the epithelium, and, in addition, could be partially the result of an uptake into the airway epithelium. Furthermore, we found elevated circulating levels of ET-1 in arterial and venous samples in COPD, and significantly higher levels in COPD with PAH. Increased circulating ET-1 levels have been recently shown in exacerbation of COPD [41], whereas there are still controversial data about ET-1 levels in the blood of patients with PAH associated with COPD or emphysema [19-21]. While a study disclosed increased levels of ET-1 only in pulmonary arterial sample [19], two reports showed persistent increased circulating ET-1 levels, which correlated to the severity of pulmonary hypertension [20,21]. In our group of COPD patients with PAH, ET-1 levels in arterial sample positively correlated with PaPs, however we provide new evidence that also ET-1 in EBC is related to PaPs. Interestingly, in the same group, we observed a negative correlation between ET-1 levels in arterial blood and FEV_1_%, but not between ET-1 levels in EBC and FEV_1_%. This finding sustains the previous hypothesis that pulmonary endothelium represents the mayor source of ET-1, which could be only in part produced by the airway epithelium, where it may contribute to induce partial bronchoconstriction, although the latter observation needs to be validated by further histological evidences. As previously demonstrated [42], an inverse correlation between PaPs and PaO_2 _was observed in this group, confirming that pulmonary artery pressure and PaO_2 _are closely negatively related in PAH induced by COPD. Regarding to exhaled NO, we found reduced levels of FENO in COPD with PAH compared to the group of COPD. Clini and co-workers previously showed reduced levels of FENO in PAH induced by COPD compared to COPD only, which negatively correlated with PaPs [27]. In our experience, FENO levels were also significantly lower in these patients, but we failed to find correlation with Echo-Doppler assessment. In summary, we observed a pathological dysregulation output in the breath between ET-1 and NO in COPD with PAH. Whether the imbalance in the release of these two mediators is an early cause or result of pulmonary hypertension due to COPD is unknown, however the easy and quick detection by a non-invasive and useful assay might encourage further investigations.

This study has different limitations, likely due to methodological assays. First, the control group consisted by healthy non smokers, and not by ex-smokers with no airflow obstruction, that would have been represented a better control group. However our COPD patients were all non smokers from at least 1 year, we consider that smoking habit might not have significantly influenced exhaled breath results. Second, in COPD patients, the pulmonary arterial pressure was evaluated by echocardiography analysis, which does not correspond to the gold standard; however in 4 patients was previously performed a right heart catheterization and the results were comparable with the echo Doppler assessment. Moreover, although no COPD patient was under steroid treatment, we could not exclude that other treatments, such as antihypertensive, nitro-derivate, or other drugs, might have influenced the results. Finally, no significant difference in the treatment between COPD and COPD with pulmonary hypertension was evident.

## Conclusion

In conclusion, we found increased expression of ET-1 in EBC and in the blood, in COPD patients with PAH compared to COPD only. ET-1 levels, both in EBC and in the arterial samples, positively correlated to pulmonary-artery systolic pressure, while arterial concentration of ET-1 negatively correlated with FEV_1_%, indicating that enhanced ET-1 levels in biological sample might early occur in PAH secondary to COPD, and actively participate in stimulating vascular and airway remodelling, through an endogenous increased production by the endothelium, and by a partial release and uptake into the airway epithelium. Finally, we showed an imbalance output in the breath between ET-1 and NO in this group of patients, suggesting that suppression of NO, in pulmonary hypertension, might have been caused in part by ET-1 [43]. Although the novel treatment for pulmonary artery hypertension has been used successfully for IPAH, it remains to be investigated with randomized multicentric studies whether this new therapy could provide beneficial clinical effects in PAH secondary to COPD.

## Abbreviations

ATS: American Thoracic Society; COPD: Chronic Obstructive Pulmonary Disease; D_L_CO: Carbon monoxide diffusion capacity; EBC: Exhaled Breath Condensate; ERS: European Respiratory Society; ET-1: Endothelin-1; FENO: Exhaled Nitric Oxide; FEV_1_: Forced Expiratory Volume in the first second; FVC: Forced Vital Capacity; HS: Healthy Subjects; NO: Nitric Oxide; PAH: Pulmonary Artery Hypertension; IPAH: Idiopathic Pulmonary Artery Hypertension; PaPs: Pulmonary-artery systolic pressure; ppb: parts per billion; RV: Residual Volume; 6MWD: 6-minutes walking distance.

## Competing interests

The authors declare that they have no competing interests.

## Authors' contributions

PC designed the study, carried out the laboratory research and the patients' characterisation for the classification of the different patient groups, and wrote the manuscript. CS designed the study, recruited the patients, coordinated the study, and assisted in performing the statistical analysis. MM performed the statistical analysis, participated in interpretation of results, in writing the manuscript, and critically reviewed the manuscript. TSM assisted in the patients' characterisation, performed laboratory analysis and interpretation of results. GDG recruited the patients and helped with study design. FG assisted in the patients' characterisation, in statistical analysis, and interpretation of results. RAC performed the statistical analysis and participated in reviewing the manuscript. SD assisted in the patients' characterisation and in statistical analysis. PP recruited the patients and helped with study design. AS participated in interpretation of results and critically reviewed the manuscript. OR conceived and supervised the study as head of the lung research group, participated in its design and coordination and revised the manuscript. All authors read and approved the final manuscript.

## Aknowledgments

The authors would like to thank Gaia Iacoviello MD, Fabio Cardinale MD, and G. Elisiana Carpagnano MD, for technical support

## Pre-publication history

The pre-publication history for this paper can be accessed here:


